# AI-supported virtual laboratory for flipped physics learning: effects on middle school students’ achievement, motivation, and cognitive load

**DOI:** 10.3389/fpsyg.2026.1939220

**Published:** 2026-10-21

**Authors:** Yuan Li, Nur Jahan Ahmad

**Affiliations:** 1 School of Physics and Electronic Engineering, Nanyang Normal University, Nanyang, China; 2 School of Educational Studies, Universiti Sains Malaysia, Penang, Malaysia

**Keywords:** AI-supported virtual laboratory, cognitive load, flipped classroom, learning achievement, learning motivation, physics learning

## Abstract

This study examined the effects of an AI-supported virtual laboratory (AI-VL) on middle school students’ flipped physics learning. A pretest–posttest control-group quasi-experimental design was adopted with 90 Grade 9 students from a middle school in central China. Three intact classes were assigned to the AI-VL, virtual laboratory (VL), and physical laboratory (PL) groups, with 30 students in each group. ANCOVA revealed large group effects on physics learning achievement, *F*(2, 86) = 113.38, *p* < 0.001, partial η^2^ = 0.73, and overall learning motivation, *F*(2, 86) = 113.37, *p* < 0.001, partial η^2^ = 0.73. The AI-VL group outperformed the VL and PL groups, while the VL group also outperformed the PL group. The AI-VL group reported higher intrinsic motivation, whereas the group difference in extrinsic motivation was not statistically significant (*p* = 0.054). Significant group differences were also found across the three cognitive load dimensions, with *F*(2, 87) values ranging from 9.07 to 72.94 and partial η^2^ values ranging from 0.17 to 0.63. The AI-VL group reported the lowest perceived intrinsic cognitive load and the highest germane cognitive load, while both AI-VL and VL resulted in lower extraneous cognitive load than PL. These findings extend research on virtual laboratories and AI-supported learning by demonstrating that AI can be embedded within a VL to help students manage cognitive demands and engage in meaningful learning. Practically, AI-VL can complement PL in flipped physics learning, particularly for pre-class preparation, review, and topics involving abstract or difficult-to-observe processes, with AI agents designed as learning guides rather than answer providers.

## 1 Introduction

STEM education is generally concerned with learning across science, technology, engineering, and mathematics and aims to develop students’ scientific understanding, problem-solving ability, and capacity to apply knowledge to real-world situations (Tan et al., 2023). Within this broader field, physics plays an important role because it helps students explain natural phenomena through concepts, models, mathematical representations, and experimental evidence (Tschisgale et al., 2023). Learning physics therefore requires more than remembering definitions or formulas. Students need to understand abstract concepts, interpret symbolic and graphical representations, connect theory with experimental observations, and apply principles to solve problems (Docktor and Mestre, 2014). These requirements make physics learning cognitively demanding, especially for middle school students who are still developing formal reasoning and scientific inquiry skills. As a result, many students experience difficulties in building coherent conceptual understanding, which may lead to unsatisfactory learning achievement, reduced motivation, and high cognitive load during physics learning (Liu et al., 2022).

Traditional physics instruction is often organized as a teacher-centered sequence in which students first learn concepts, laws, and formulas through classroom explanation and then conduct laboratory activities to verify what has been taught. Although this approach provides structured instruction, it may not fully address the challenges students face in physics learning, particularly when laboratory activities emphasize procedural completion rather than conceptual understanding (Oliveira and Bonito, 2023). When theoretical knowledge is presented before students have sufficient opportunities to explore phenomena, laboratory work may become a process of confirming known results rather than a means of constructing conceptual understanding. Students may remain passive recipients of information, and their misconceptions or learning difficulties may not be identified in time. In addition, physical laboratory activities are often constrained by equipment availability, laboratory space, safety requirements, and fixed schedules, limiting opportunities for repeated practice and self-paced inquiry (Wang et al., 2024). Oliveira and Bonito’s (2023) systematic review found that practical work in science education can support conceptual understanding, but its effectiveness depends on meaningful task design and adequate teacher guidance. Their review also showed that practical activities may lose educational value when students focus mainly on completing procedures rather than understanding scientific ideas. Therefore, more effective instructional approaches are needed to address students’ difficulties in physics learning.

The flipped classroom has been regarded as a useful approach for improving students’ active learning in science education. In this approach, direct instruction is moved before class through videos, readings, or other learning materials, while classroom time is used for problem-solving, discussion, inquiry, and teacher-supported activities (Chen et al., 2026). This structure can give students more time to review basic content before class and allows teachers to use class time for deeper learning activities. In physics education, the flipped classroom has been reported to support students’ learning achievement and motivation by shifting students from passive listening to more active engagement with concepts and problems (Chen et al., 2025). For example, Tunggyshbay et al. (2023) reviewed 30 studies on flipped classrooms in physics education and found that this approach generally had positive effects on students’ learning outcomes compared with traditional classroom instruction. However, the effectiveness of a flipped classroom depends strongly on the quality of pre-class learning and students’ engagement with learning materials. Lo and Hew (2017) noted that students may enter class underprepared when pre-class materials are not sufficiently clear, engaging, or well aligned with in-class activities. A flipped classroom also requires students to manage their learning before class, which can be difficult for learners with weak self-regulation. More importantly, videos and textbooks alone may not provide sufficient opportunities for students to observe phenomena, manipulate variables, test ideas, and connect theoretical explanations with experimental evidence (Tunggyshbay et al., 2023). Therefore, flipped physics learning may benefit from technology-supported environments that provide interactive inquiry and timely learning support (Errabo et al., 2024; Lo and Hew, 2017).

With the development of educational technology, virtual laboratory (VL) has become an important learning environment for science and physics education. VL allows students to conduct experiments through simulations, digital models, and interactive operations, making it possible to observe phenomena, manipulate variables, and repeat experimental procedures without being fully constrained by laboratory space, equipment, or safety conditions (May et al., 2023). Recent reviews in physics education suggest that virtual experiments can help address common difficulties in physical experiments, including limited equipment, restricted experimental conditions, and insufficient opportunities for repeated practice (Wang et al., 2024). In flipped learning, VL can further extend pre-class learning beyond videos and textbooks by giving students opportunities to explore experimental phenomena before or outside classroom instruction (Agusty and Chen, 2026). For example, Errabo et al. (2024) showed that integrating virtual laboratories into flipped classroom delivery can support scientific enquiry, problem-solving, metacognition, self-efficacy, and students’ laboratory performance. However, the use of VL alone may not fully support students when they encounter difficulties during self-paced pre-class learning. Students may still misunderstand scientific concepts, follow procedures mechanically, or fail to interpret experimental results correctly (Chen et al., 2025; Chen et al., 2026). In addition, it is difficult for one teacher to monitor each student’s learning process and respond to every individual problem in a timely manner, especially when students learn at their own pace before class. Therefore, how to provide timely feedback and learning support when students encounter problems during pre-class physics learning remains an important issue.

The rapid development of artificial intelligence (AI) provides a possible way to address this problem. AI-supported learning systems can provide adaptive guidance, personalized explanations, automated feedback, and question-answering support based on students’ learning needs (Xu and Ouyang, 2022). These functions are particularly relevant to self-paced learning contexts, in which students may require immediate assistance when direct teacher support is unavailable. Recent studies have demonstrated the application of AI-supported tools in physics and science learning, including intelligent tutoring, chatbot-supported explanation, adaptive feedback, and worked-example support (Chen and Chang, 2024; Kestin et al., 2025; Lademann et al., 2025). Nevertheless, research examining the integration of AI support directly within virtual laboratories, particularly in flipped secondary physics learning, remains limited (Xu and Ouyang, 2022). To address this gap, the present study designed an AI-supported virtual laboratory (AI-VL) and applied it to a middle school flipped physics learning classroom context. This study compared three learning conditions: the AI-supported virtual laboratory group, the virtual laboratory group (VL), and the physical laboratory group (PL). It examined whether AI-VL could improve students’ learning achievement and motivation and whether it could reduce cognitive load during flipped physics learning. The following research questions guided the study:

RQ1: Does AI-VL improve middle school students’ physics learning achievement compared with VL and PL?

RQ2: Does AI-VL enhance middle school students’ learning motivation compared with VL and PL?

RQ3: Are there significant differences in cognitive load among students in AI-VL, VL, and PL?

## 2 Literature review

### 2.1 Flipped classroom in physics learning

The flipped classroom is an instructional approach in which students first learn basic content before class through videos, readings, or other learning materials and then use classroom time for discussion, problem-solving, inquiry, and teacher-guided activities (Chen et al., 2026). In physics learning, this model is particularly relevant because students often require repeated exposure to abstract concepts before applying them in experiments or problem situations. By moving direct instruction outside the classroom, the flipped classroom can provide more time for active learning and allow teachers to address students’ conceptual difficulties during class (Lo and Hew, 2017).

A growing body of research has examined flipped classroom practices in physics education. Tunggyshbay et al. (2023) conducted a systematic review showed that flipped physics learning was often supported by videos, online platforms, simulations, collaborative tasks, and inquiry-based activities. This finding suggests that flipped classroom in physics is not simply a change in the order of instruction, but a learning design that depends on the quality of pre-class preparation and in-class activities. Empirical studies have also reported positive effects of flipped classroom on physics learning achievement and motivation. Cagande and Jugar (2018) used a Solomon four-group design to examine the effects of a flipped classroom intervention on college physics students’ motivation and understanding of kinematics graphs. They found that the flipped classroom improved students’ conceptual understanding and supported their learning motivation. Aşıksoy and Özdamlı (2016) applied a flipped classroom approach based on the ARCS motivation model in a physics course and found positive effects on students’ academic achievement, motivation, and self-efficacy. These findings indicate that flipped physics learning can support both cognitive and motivational outcomes when students are given structured pre-class materials and meaningful learning activities.

However, the flipped classroom also places high demands on students and teachers. Akçayır and Akçayır (2018) conducted a systematic review of 71 studies published in SSCI-indexed journals and found that although flipped classroom was often associated with improved learning performance, several challenges were concentrated in out-of-class learning activities. These challenges included inadequate student preparation before class, limited engagement with pre-class materials, and problems related to the quality of video-based learning resources. These issues are especially relevant to physics learning because students may not fully understand abstract concepts through videos and textbooks alone. Without sufficient guidance and feedback during the pre-class stage, students may enter class with unresolved misconceptions and incomplete understanding. Therefore, flipped physics learning may require additional learning environments that can support students’ preparation, inquiry, and feedback needs before class.

### 2.2 Virtual laboratory in physics learning

Virtual laboratories (VLs) have been increasingly used in physics education because they can provide interactive, visual, and repeatable experimental experiences. In a VL environment, students can manipulate variables, observe changes, test hypotheses, and repeat experimental procedures without being fully constrained by laboratory space, equipment availability, or safety conditions. These features are particularly relevant to physics learning, in which many concepts and processes are abstract or difficult to observe directly using physical apparatus (Finkelstein et al., 2005).

Empirical studies have shown that VL can support students’ physics learning in ways that are comparable to or, in some cases, stronger than PL. Al-Duhani et al. (2023) compared virtual and physical laboratory instruction in Grade 8 electricity learning and found that students in the virtual laboratory group achieved higher learning scores. Their findings suggest that virtual laboratories can support the understanding of electricity-related concepts by providing repeatable operations and clearer representations of abstract processes. Papalazarou et al. (2023) compared students’ learning of physics concepts in virtual and physical inquiry-based experiments and found no significant difference in conceptual improvement between the two laboratory modes. They also noted that VL appeared to have advantages for electricity-related topics because it helped students visualize invisible elements and focus on conceptual relationships. Recent studies have also examined more immersive forms of virtual laboratory learning. Sun et al. (2023) used a quasi-experimental design to investigate wearable AR and hybrid AR/VR materials in a high school physics laboratory environment. They found that the hybrid AR/VR group showed higher situational interest, engagement, and learning performance than the traditional learning group. Similarly, Korlat et al. (2024) examined a game-based virtual reality laboratory for secondary school physics and found that students in the experimental group achieved higher scores on a later physics test than those in the control group. These studies indicate that technology-enhanced laboratory environments can support both learning performance and motivational outcomes in physics education. Review evidence further supports the educational value of VL in physics learning. Review evidence further indicates that virtual experiments can address limitations related to equipment, experimental conditions, and opportunities for repeated practice. However, their educational effectiveness remains dependent on appropriate instructional design, guidance, and conceptual support (Wang et al., 2024). Without sufficient guidance, students may use VL mainly to complete procedures rather than to build a deeper understanding of physics concepts. Therefore, while VL can extend students’ opportunities for physics experimentation, its effectiveness depends on whether students receive adequate guidance, feedback, and conceptual support during the learning process.

### 2.3 AI-supported STEM learning

Artificial intelligence (AI) has been increasingly integrated into STEM education to provide adaptive guidance, personalized explanations, automated feedback, and question-answering support. Unlike static learning materials, AI-supported systems can respond to students’ questions, provide immediate explanations, and support learners when teachers are not available. These functions are useful in STEM learning, where students often need help with abstract concepts, problem-solving steps, procedural errors, and the interpretation of scientific representations (Ait Baha et al., 2024).

Recent empirical studies have reported positive effects of AI-supported learning in STEM-related contexts. Kestin et al. (2025) conducted a randomized controlled trial in an undergraduate physics course and compared an AI-powered tutor with an active learning class. They found that students using the AI tutor achieved higher learning gains in less time and reported higher engagement and motivation than students in the active learning condition. Lademann et al. (2025) examined AI custom chatbots in mathematics and physics learning with sixth-grade students. Their study found that AI-generated explanations improved positive activating emotions, situational interest, and self-efficacy, while reducing intrinsic and extraneous cognitive load. Becker et al. (2025) further showed that AI chatbot scaffolding in physics learning was associated with positive affective outcomes, including enjoyment, self-efficacy, and situational interest. This finding suggests that AI-based support may influence not only cognitive outcomes but also students’ motivational and emotional engagement with physics learning.

AI-supported learning has also been examined in school science and broader STEM learning. Chen and Chang (2024) used an experimental design to investigate AI-assisted game-based learning among seventh-grade students. They found that students who received ChatGPT support with worked examples achieved better science learning outcomes, showed stronger perceived competence, and reported lower mental burden than students in the game-only condition. Lin et al. (2025) developed a GPT-assisted summarization tool for STEM collaborative learning and found that students using the tool showed better learning outcomes and reflective thinking than those in the control group.

### 2.4 Research gaps and the present study

The literature reviewed above has developed largely along two parallel lines. Research on VL has demonstrated their potential to support visualization, repeated experimentation, learning achievement, and motivation in physics education. However, it has also emphasized the need for timely guidance and conceptual support during independent learning. In contrast, research on AI-supported STEM learning has shown that AI tutors, chatbots, and other AI tools can provide immediate explanations and feedback, but these tools have generally been examined as standalone learning systems rather than as integrated components of virtual experimental environments.

Three research gaps can therefore be identified. First, limited empirical evidence is available on the integration of AI support directly within a virtual laboratory, particularly in flipped secondary physics learning. Second, previous studies have rarely employed a three-group design that distinguishes the additional contribution of AI support from the effect of the virtual laboratory itself. Consequently, it remains unclear whether observed benefits arise from the virtual experimental environment, the embedded AI support, or their combination. Third, relatively few studies have simultaneously examined learning achievement, motivation, and different dimensions of cognitive load when evaluating AI-supported virtual laboratories.

To address these gaps, the present study compared an AI-supported VL with the same VL without AI support, as well as a PL, in a Grade 9 flipped physics learning environment. This three-group design distinguished the contribution of the AI agent from that of the VL environment. By examining physics learning achievement, learning motivation, and intrinsic, extraneous, and germane cognitive load, the study provides a more comprehensive evaluation of AI-supported VL learning.

## 3 Design and functions of the AI-supported virtual laboratory

The AI-supported virtual laboratory (AI-VL) used in this study was designed and implemented on the NOBOOK platform. NOBOOK provides a teacher-side interface for designing and releasing virtual physics experiments and a student-side interface for completing learning activities. The platform supports both iOS and Android systems, and students in this study mainly accessed the AI-VL through mobile devices. The teacher designed the experimental tasks through the teacher interface and released them to students, while students accessed and completed the tasks through the student interface. As shown in Figure 1, the AI-VL consisted of three internally connected databases: a learning materials database, an experimental materials database, and an AI support database. These databases were linked to support students’ access to learning resources, virtual experimental materials, and AI-based learning assistance within the same learning environment. The selected learning topic was a Grade 9 physics experiment on the relationship between current and resistance under constant voltage in Ohm’s law. The topic was selected because it is a core component of the Grade 9 physics curriculum and requires students to connect abstract electrical concepts with experimental observations. The relationship between current and resistance involves invisible processes and coordinated manipulation of multiple circuit components, which can be difficult for students to understand through explanation alone.

**Figure 1:**
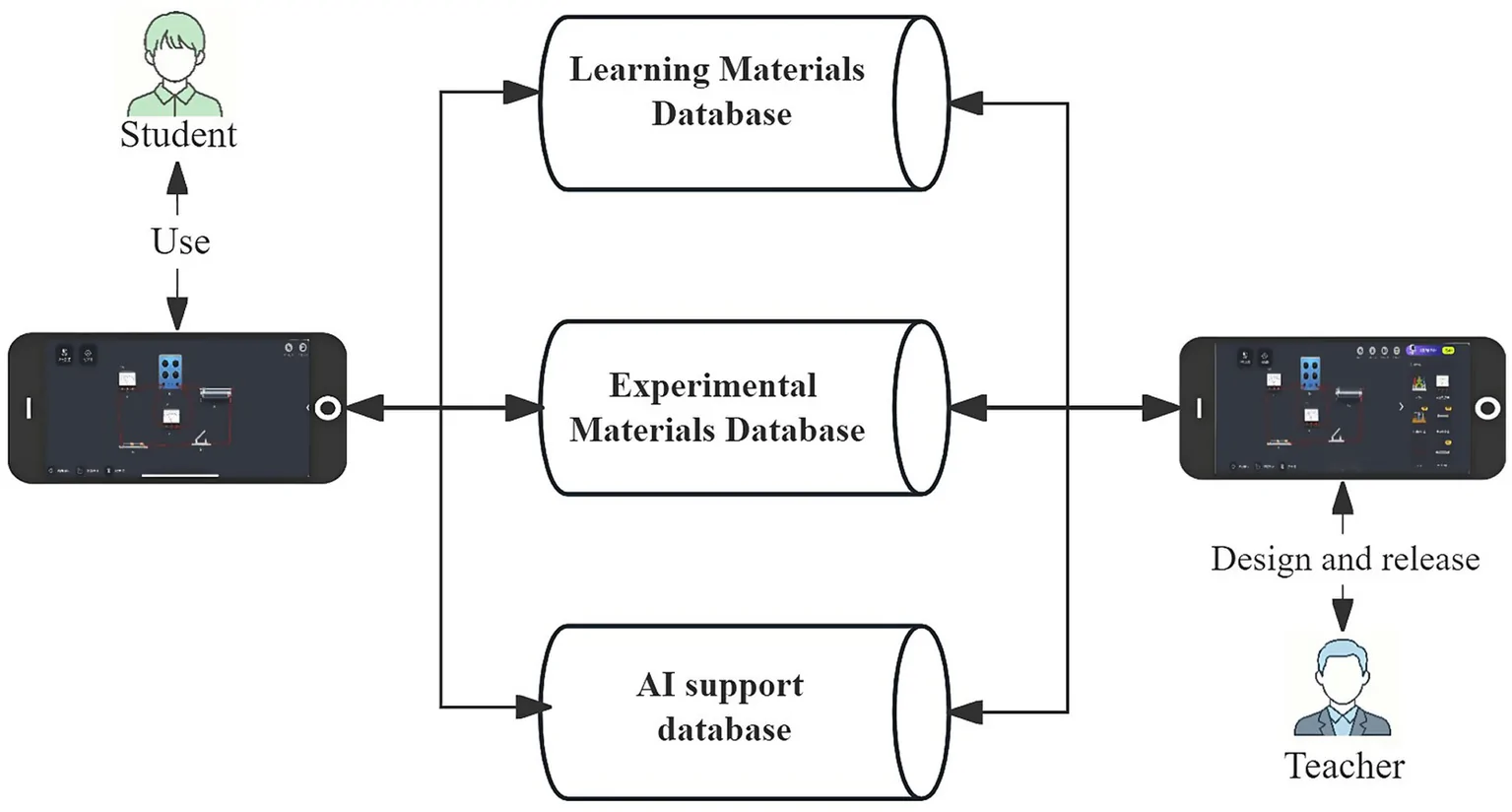
Architecture of the AI-supported virtual laboratory.

### 3.1 Learning materials database

The learning materials database provided students with the resources needed for pre-class preparation, including learning videos, experiment guides, and experimental procedures. These materials helped students understand the learning objectives, become familiar with the experimental process, and prepare for the virtual experiment during the one-week flipped learning period. As shown in Figure 2, the learning video demonstrated the experiment on the relationship between current and resistance under constant voltage.

**Figure 2:**
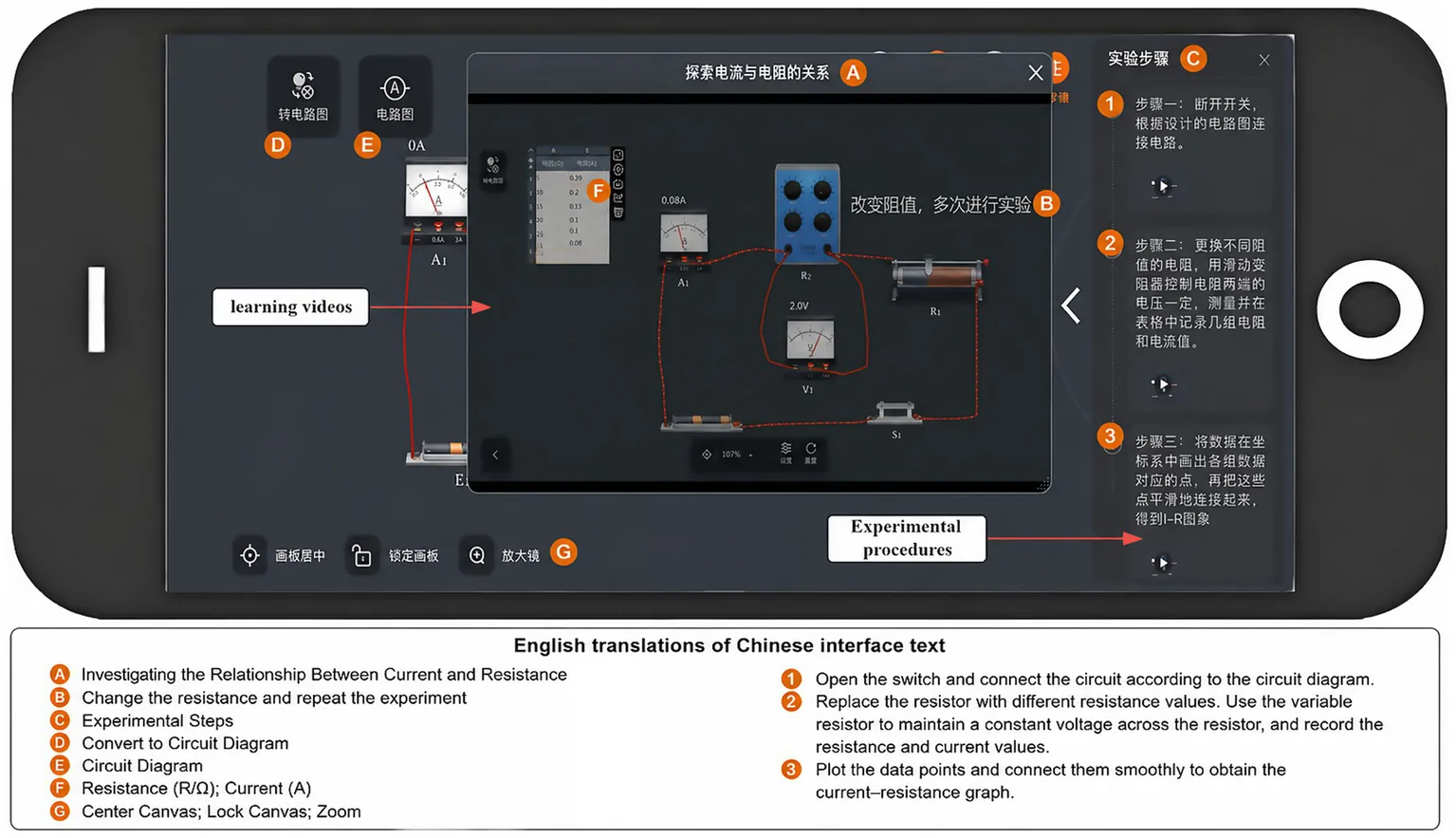
Learning video in the learning materials database.

### 3.2 Experimental materials database

The experimental materials database provided the virtual apparatus required for the physics experiment, including the resistance box, resistor, ammeter, voltmeter, switch, power supply, and connecting wires. As shown in Figure 3, students could use these apparatuses to construct the circuit and complete the experimental task in the virtual environment. The database also supported interactive operations, such as connecting wires, adjusting resistance values, opening or closing the switch, and reading data from the meters. These functions enabled students to conduct the experiment through direct manipulation and observation.

**Figure 3:**
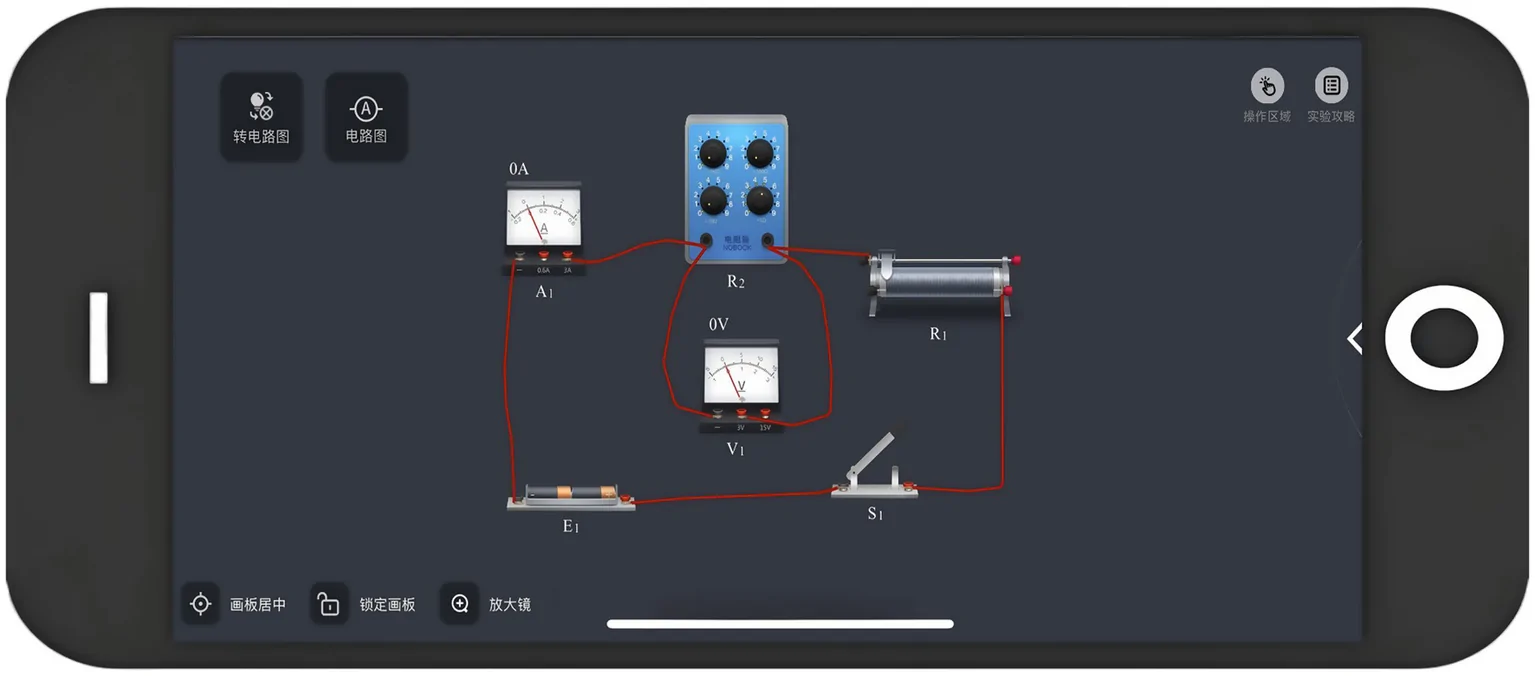
Virtual apparatus and interactive operations in the experimental materials database.

### 3.3 AI support database

The AI support database integrated a text-based conversational agent into the AI-VL to provide learning assistance during the experiment. According to the technical information provided by NOBOOK, the AI agent was built on an education-customized DeepSeek large language model developed through collaboration between DeepSeek and NOBOOK. The model was adapted for K–12 science education through education-oriented instruction tuning, domain-specific prompt engineering, and retrieval-augmented generation (RAG) based on a K–12 science knowledge base. These configurations enabled the AI agent to provide explanations of experimental principles, answer questions, analyze errors, and generate inquiry-oriented prompts relevant to middle school physics. The AI agent supported text-based interaction only and did not accept image input. Its responses were constrained to the relevant curriculum content and designed to guide students’ reasoning rather than provide final answers directly.

The AI agent provided two modes of interaction. In the first mode, students could select common preset questions and receive corresponding guidance, as shown in Figure 4. In the second mode, students could enter their own questions and obtain more targeted explanations or feedback, as shown in Figure 5. These two modes were intended to address both common learning difficulties and individual problem-solving needs during the flipped learning process.

**Figure 4:**
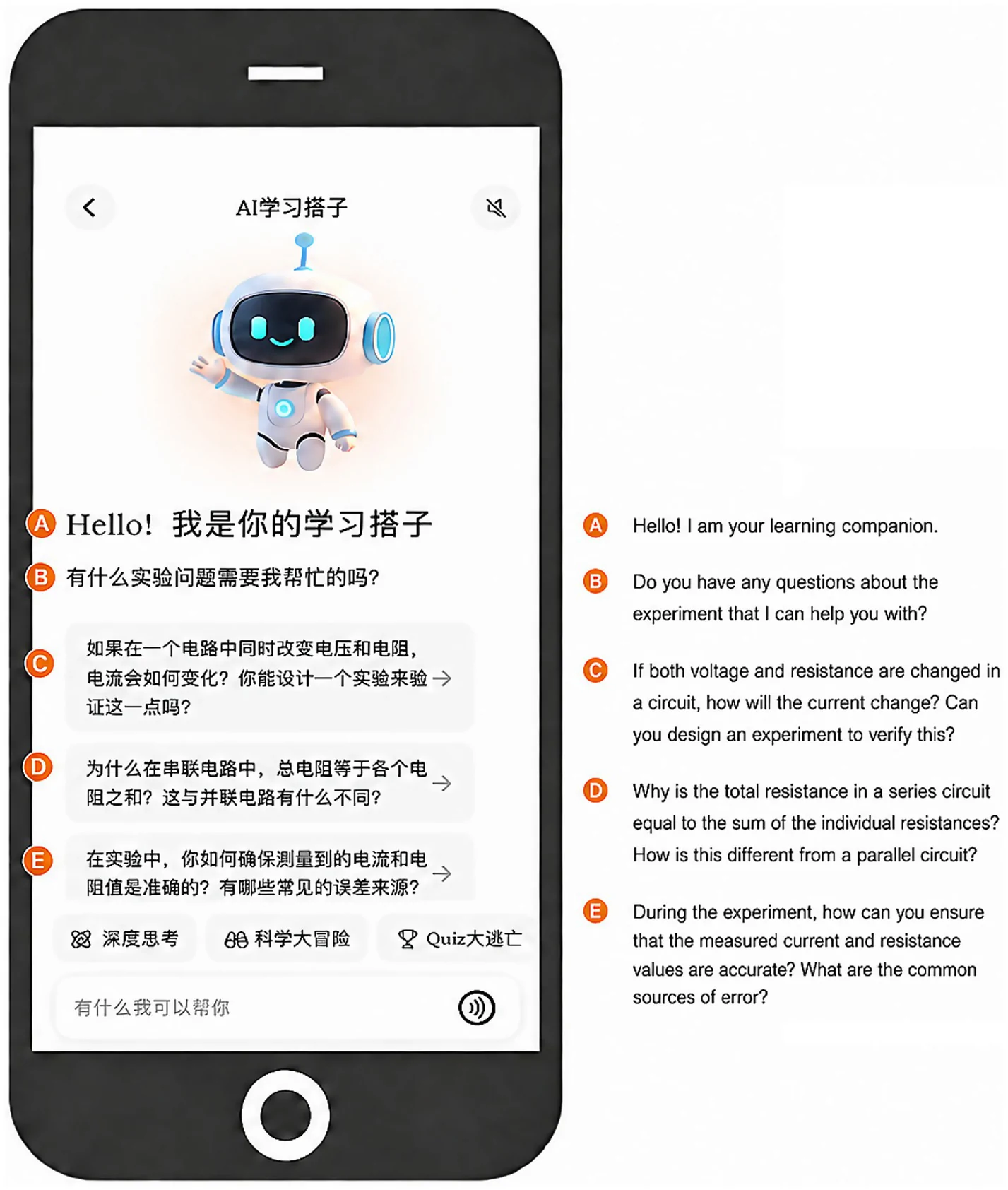
Preset question mode in the AI support database.

**Figure 5:**
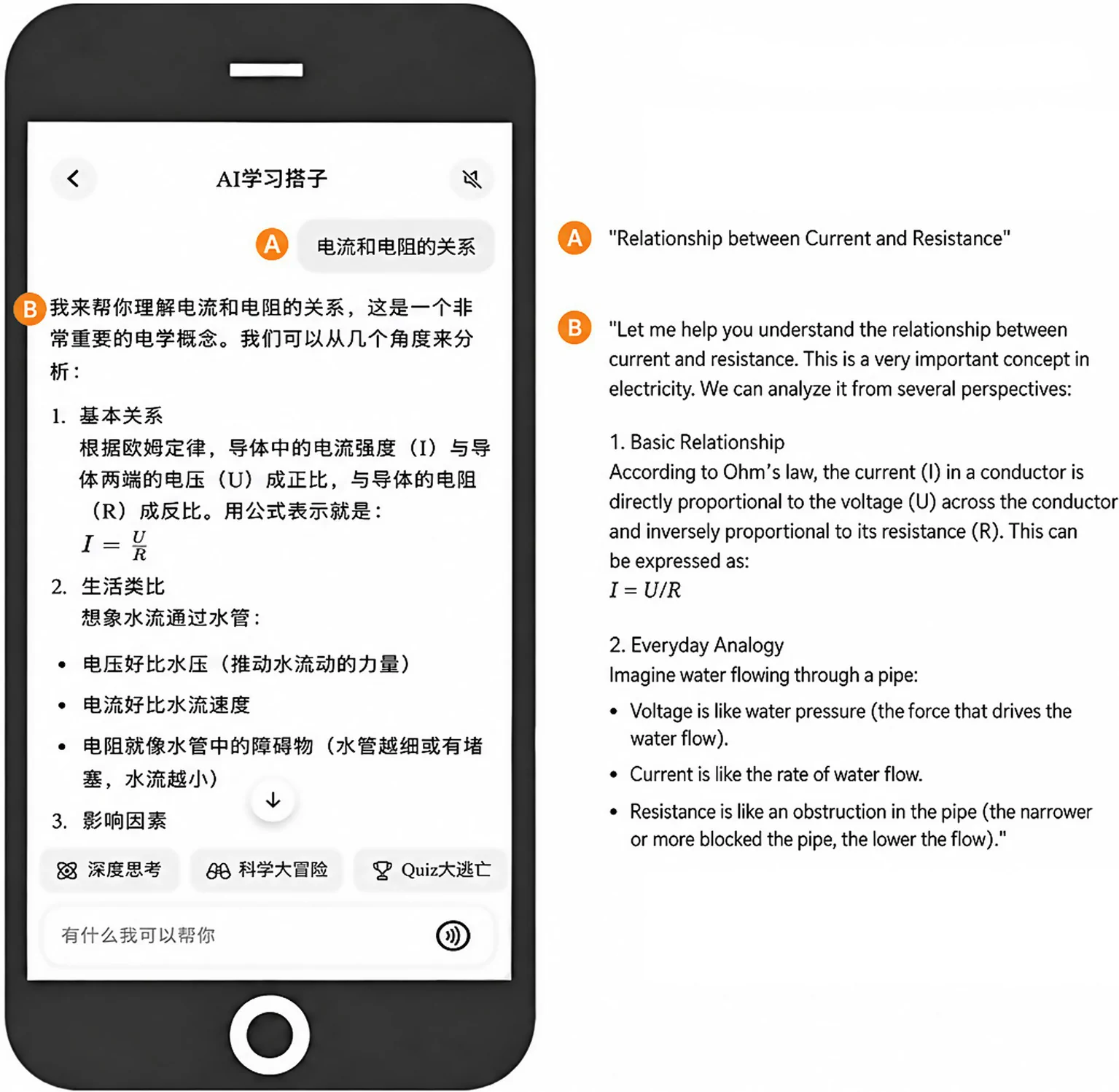
Text-based interaction mode in the AI support database.

### 3.4 Alpha and Beta testing

Before the formal intervention, alpha and beta testing were conducted to examine the usability and instructional appropriateness of the AI-VL. Alpha testing was conducted with three experts in educational technology, each with at least 8 years of professional experience. The experts were invited to review the system in terms of content accuracy, instructional design, interface usability, experimental procedures, and the appropriateness of AI-supported feedback. Based on their comments, minor revisions were made to improve the clarity of learning materials, the sequence of experimental operations, and the wording of AI prompts.

After the alpha testing, beta testing was conducted with 20 Grade 9 students from another school who did not participate in the formal experiment. The students were asked to use the AI-VL to complete the same physics learning task. The purpose of beta testing was to examine whether students could understand the learning instructions, operate the virtual apparatus, interact with the AI agent, and complete the experimental task without major difficulties. Students’ feedback was used to further refine the system interface and task instructions before the formal study.

## 4 Materials and methods

### 4.1 Research design

This study adopted a pretest–posttest control group quasi-experimental design to examine the effects of the AI-supported virtual laboratory on middle school students’ flipped physics learning. Grade 9 students were assigned to three groups: the AI-supported virtual laboratory group (AI-VL), the virtual laboratory group (VL), and the physical laboratory group (PL). The AI-VL group used the AI-supported virtual laboratory, the VL group used the same virtual laboratory without AI support, and the PL group used the physical laboratory.

### 4.2 Participants

Ninety Grade 9 students from a middle school in central China participated in this study. The participants were selected through one-stage cluster sampling. Specifically, three intact classes were selected from eight Grade 9 classes in the school and then randomly assigned to the three learning conditions: the AI-supported virtual laboratory group (AI-VL), the virtual laboratory group (VL), and the physical laboratory group (PL). Each group included 30 students. The students were between 14 and 15 years old (M = 14.52, SD = 0.50). The three classes were taught by physics teachers with at least 8 years of teaching experience. To reduce differences caused by instruction, all teachers were asked to follow the same learning objectives, experimental procedures, learning materials, experimental tasks, and assessment schedule.

Ethical considerations were addressed before the study. First, written informed consent was obtained from students’ parents or legal guardians, and students were informed that their participation was voluntary. They were allowed to withdraw from the study at any time without any negative consequences. Second, the test scores collected for the study were used only for research purposes and were not included in students’ final course grades. Third, students’ personal information was kept confidential, and all data were anonymized before analysis. Finally, to reduce concerns about unequal access to learning resources, the AI-VL was made available to all participating students after the experiment. It was also later introduced to all Grade 9 students in the school. The study was conducted in accordance with the school’s requirements for educational research involving students.

### 4.3 Instruments

This study used three instruments to answer the research questions. Physics learning achievement was measured using a pretest and posttest achievement test to answer RQ1. Learning motivation was measured using a pretest and posttest motivation scale to answer RQ2. Cognitive load was measured using a post-intervention cognitive load scale to answer RQ3.

#### 4.3.1 Physics learning achievement test (PLAT)

Students’ physics learning achievement was assessed using a pretest and a posttest developed for the target learning topic. The test focused on the Grade 9 physics experiment concerning the relationship between current and resistance under constant voltage in Ohm’s law. The items were developed with reference to Bloom et al.’s (1956) taxonomy of educational objectives and covered four cognitive levels: knowledge, comprehension, application, and analysis.

The test contained 10 multiple-choice items and 5 fill-in-the-blank items. Each of these items was scored 1 point, giving a subtotal of 15 points for assessing students’ basic knowledge and conceptual understanding. In addition, the test included 2 calculation items, each worth 5 points, to examine students’ application and analytical skills. A further analysis item, worth 5 points, was included to assess students’ deeper reasoning about the experimental content. The total score therefore ranged from 0 to 30, with higher scores representing better physics learning achievement.

Content validity was examined through expert review. One professor of physics and 2 physics teachers, each with more than 8 years of teaching experience, evaluated the relevance of the test items using the Item Content Validity Index (I-CVI). Items with lower relevance ratings were revised according to the experts’ comments. The Scale-level Content Validity Index (S-CVI) was 0.93 for the pretest and 0.96 for the posttest, indicating strong content validity. The final version of the achievement test was confirmed after all experts approved the revised items.

#### 4.3.2 Physics learning motivation scale (PLMS)

Students’ physics learning motivation was measured using the Physics Learning Motivation Scale (PLMS) adopted from Chen et al. (2025). The PLMS was administered as both a pretest and a posttest. It consists of 10 items rated on a 5-point Likert scale, ranging from 1 = strongly disagree to 5 = strongly agree. The total score ranges from 10 to 50, with higher scores indicating stronger physics learning motivation.

The scale includes two dimensions: intrinsic motivation and extrinsic motivation. Items 1–5 measure intrinsic motivation, such as “I think learning physics is interesting because it improves my knowledge” and “I find satisfaction in understanding physics concepts deeply.” Items 6–10 measure extrinsic motivation, such as “I study physics because it could improve my future career prospects” and “My motivation to study physics is influenced by my parents’ expectations.” Chen et al. (2025) reported good internal consistency for the PLMS, with Cronbach’s α = 0.86 for the full scale, α = 0.85 for intrinsic motivation, and α = 0.88 for extrinsic motivation. In the present study, Cronbach’s alpha was 0.88 for the overall PLMS, 0.84 for the intrinsic motivation subscale, and 0.86 for the extrinsic motivation subscale, indicating good internal consistency.

#### 4.3.3 Cognitive load scale (CLS)

Students’ cognitive load was measured after the intervention using the Cognitive Load Scale (CLS) adapted from Leppink et al. (2013). The original scale consists of 10 items measuring three types of cognitive load: intrinsic cognitive load, extraneous cognitive load, and germane cognitive load. Items 1–3 measure intrinsic cognitive load, such as the perceived complexity of the topic, formulas, concepts, and definitions. Items 4–6 measure extraneous cognitive load, focusing on the clarity and effectiveness of the instructions and explanations. Items 7–10 measure germane cognitive load, reflecting the extent to which the learning activity enhanced students’ understanding of the topic, formulas, concepts, and definitions.

In the original scale, students responded on an 11-point scale ranging from 0 to 10. In this study, a 5-point Likert scale was used to reduce the response burden for middle school students, ranging from 1 = strongly disagree to 5 = strongly agree. For intrinsic cognitive load and extraneous cognitive load, lower scores indicate a lower perceived level of content complexity and unnecessary learning difficulty. For germane cognitive load, higher scores indicate greater cognitive engagement in understanding and organizing the learning content. Leppink et al. (2013) reported acceptable to high internal consistency for the three subscales, with Cronbach’s α values ranging from 0.81 to 0.88 for intrinsic cognitive load, 0.75 to 0.85 for extraneous cognitive load, and 0.82 to 0.93 for germane cognitive load. For this study, Cronbach’s alpha values were 0.86 for intrinsic cognitive load, 0.82 for extraneous cognitive load, and 0.88 for germane cognitive load, indicating good internal consistency.

### 4.4 Experimental procedure

The experiment lasted for 3 weeks, as shown in Figure 6. In Week 1, students in the three groups first completed the pretest of the Physics Learning Achievement Test (PLAT), which lasted 40 min, and the pretest of the Physics Learning Motivation Scale (PLMS), which lasted 15 min. After the pretests, the teacher introduced the syllabus, learning objectives, experimental requirements, and learning procedures for 20 min.

**Figure 6:**
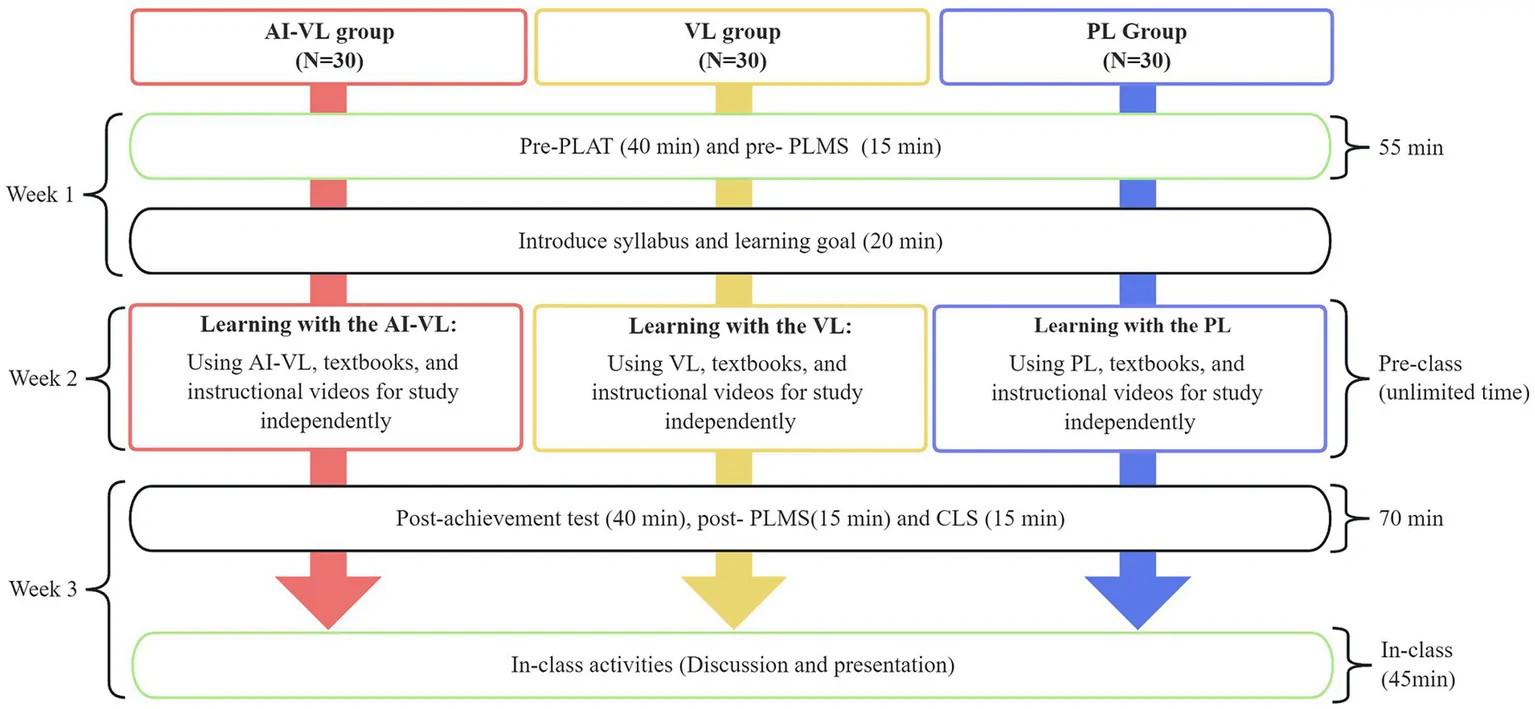
Experimental procedure.

In Week 2, students in the three groups completed the flipped learning activities under different learning conditions. All students were provided with the same textbooks and instructional videos for independent learning. The AI-VL group used the AI-supported virtual laboratory, textbooks, and instructional videos. The VL group used the same virtual laboratory, textbooks, and instructional videos, but without AI support. Thus, the AI-VL and VL groups had the same learning content and virtual experimental environment, and the main difference was whether AI-based learning assistance was available. The PL group used the physical laboratory, textbooks, and instructional videos. During this week, students in all groups were allowed to learn independently according to their own needs and schedules.

In Week 3, students completed the posttest of the PLAT, which lasted 40 min, the posttest of the PLMS, which lasted 15 min, and the Cognitive Load Scale (CLS), which lasted 15 min. These post-intervention measurements took 70 min in total. After the posttests, all groups participated in in-class activities for 45 min, including group discussion and presentation.

## 5 Data analysis and results

All quantitative data were analyzed using SPSS 26.0. To examine group differences in physics learning achievement and learning motivation, a one-way analysis of covariance (ANCOVA) was conducted with the corresponding pretest scores used as covariates. *Post hoc* comparisons were then performed to identify specific differences among the AI-VL, VL, and PL groups. A one-way analysis of variance (ANOVA) was used for cognitive load because it was measured only after the intervention. *Post hoc* comparisons were also conducted to compare the three groups in terms of intrinsic, extraneous, and germane cognitive load. The significance level was set at 0.05 for all statistical analyses.

To provide a manipulation check for the AI-VL condition, descriptive usage data for the AI agent were added. As shown in Table 1, students in the AI-VL group used the AI agent an average of 40 times, with the number of uses ranging from 28 to 56. The average AI agent use time was 42.35 min, ranging from 33.45 to 60.26 min. These results indicate that students in the AI-VL group did interact with the AI agent during the learning process.

**Table 1:** Descriptive usage data for the AI agent in the AI-VL group.

Indicator	*N*	Mean	Min	Max
Number of AI agent uses (Times)	30	40	28	56
AI agent use time (min)	30	42.35	33.45	60.26

### 5.1 Baseline comparisons

Before conducting the main analysis, one-way analyses of variance (ANOVAs) were performed to examine baseline differences among the AI-VL, VL, and PL groups in physics learning achievement and learning motivation. As shown in Table 2, no significant difference was found among the three groups in pretest physics learning achievement, *F*(2, 87) = 0.79, *p* = 0.46. However, the groups differed significantly in overall learning motivation at baseline, *F*(2, 87) = 3.73, *p* = 0.03. Bonferroni *post hoc* comparisons indicated that the PL group had significantly higher baseline learning motivation than the AI-VL group (*p* = 0.027), whereas no significant differences were found between the AI-VL and VL groups or between the VL and PL groups. No significant baseline differences were observed in intrinsic motivation, *F*(2, 87) = 1.44, *p* = 0.24, or extrinsic motivation, *F*(2, 87) = 2.37, *p* = 0.10. Given the significant baseline difference in overall learning motivation, and to control for pre-existing variation in the outcome measures, the subsequent analyses of posttest physics learning achievement and learning motivation were conducted using ANCOVA, with the corresponding pretest scores used as covariates.

**Table 2:** Baseline comparisons of pretest physics learning achievement and learning motivation among the three groups.

Variable	Group	*N*	*Mean*	*SD*	*F*	*p*	*Post hoc*
Physics learning achievement	AI-VL	30	12.77	1.65	0.79	0.46	
	VL	30	12.50	1.55			
	PL	30	12.27	1.41			
Learning motivation	AI-VL	30	3.46	0.27	3.73*	0.03	(3)>(1)
	VL	30	3.52	0.26			
	PL	30	3.65	0.28			
Intrinsic motivation	AI-VL	30	2.81	0.27	1.44	0.24	
	VL	30	2.87	0.30			
	PL	30	2.93	0.30			
Extrinsic motivation	AI-VL	30	4.12	0.43	2.37	0.10	
	VL	30	4.18	0.46			
	PL	30	4.37	0.48			

*p < 0.05.

### 5.2 Results of physics learning achievement

Before conducting the ANCOVA, the assumptions of homogeneity of variance and homogeneity of regression slopes were examined. Levene’s test showed that the assumption of homogeneity of variance was met, *F* = 1.49, *p* = 0.23. The test of homogeneity of regression slopes was also nonsignificant, *F* = 0.38, *p* = 0.69, indicating that the relationship between the pretest and posttest scores did not differ significantly across the three groups. Therefore, ANCOVA was appropriate for analyzing students’ physics learning achievement.

The ANCOVA results showed a significant difference in posttest physics learning achievement among the three groups after controlling for pretest scores, *F*(2, 86) = 113.38, *p* < 0.001, partial η^2^ = 0.73, as shown in Table 3. The adjusted mean score was highest for the AI-VL group (adjusted M = 23.45, SE = 0.32), followed by the VL group (adjusted M = 20.51, SE = 0.32) and the PL group (adjusted M = 16.57, SE = 0.32). The partial η^2^ value indicated a large effect, suggesting that the learning condition explained a substantial proportion of the variance in posttest achievement after controlling for pretest scores. Bonferroni *post hoc* comparisons showed that the AI-VL group scored significantly higher than both the VL group and the PL group. The VL group also scored significantly higher than the PL group.

**Table 3:** ANCOVA results for physics learning achievement.

Group	*N*	*Mean*	*SD*	*Adj.M*	*SE*	*F*	partial η^2^	*Post hoc*
(1) AI-VL	30	23.67	2.02	23.45	0.32	113.38***	0.73	(1) > (2)
(2) VL	30	20.50	2.61	20.51	0.32			(1) > (3)
(3) PL	30	16.37	1.83	16.57	0.32			(2) > (3)

***p < 0.001.

### 5.3 Results of physics learning motivation

Before conducting the ANCOVA, the assumptions of homogeneity of variance and homogeneity of regression slopes were examined for learning motivation and its two dimensions. For overall learning motivation, Levene’s test was nonsignificant, *F* = 1.58, *p* = 0.21 > 0.05, and the test of homogeneity of regression slopes was also nonsignificant, *F* = 3.05, *p* = 0.06 > 0.05. For intrinsic motivation, Levene’s test was nonsignificant, *F* = 1.14, *p* = 0.33 > 0.05, and the test of homogeneity of regression slopes was nonsignificant, *F* = 0.38, *p* = 0.68 > 0.05. For extrinsic motivation, Levene’s test was nonsignificant, *F* = 0.01, *p* = 0.98 > 0.05, and the test of homogeneity of regression slopes was nonsignificant, *F* = 0.23, *p* = 0.79 > 0.05. These results indicated that the assumptions for ANCOVA were met.

As shown in Table 4, the ANCOVA results showed a significant difference in overall learning motivation among the three groups after controlling for pretest motivation scores, *F*(2, 86) = 113.37, *p* < 0.001, partial η^2^ = 0.73. The adjusted mean score was highest for the AI-VL group (adjusted M = 4.67, SE = 0.03), followed by the VL group (adjusted M = 4.31, SE = 0.03) and the PL group (adjusted M = 3.94, SE = 0.03). The partial η^2^ value indicated a large effect, suggesting that the learning condition explained a substantial proportion of the variance in overall learning motivation after controlling for pretest scores. Bonferroni *post hoc* comparisons showed that the AI-VL group reported significantly higher overall learning motivation than both the VL group and the PL group. The VL group also reported significantly higher motivation than the PL group.

**Table 4:** ANCOVA results for physics learning motivation.

Variable	Group	*N*	Mean	*SD*	*Adj.M*	*SE*	*F*	partial η^2^	*Post hoc*
Learning motivation	(1) AI-VL	30	4.60	0.36	4.67	0.03	113.37***	0.73	(1) > (2)
	(2) VL	30	4.29	0.28	4.31	0.03			(1) > (3)
	(3) PL	30	4.03	0.26	3.94	0.03			(2) > (3)
Intrinsic motivation	(1) AI-VL	30	4.71	0.42	4.77	0.06	132.59***	0.76	(1) > (2)
	(2) VL	30	4.02	0.45	4.02	0.06			(1) > (3)
	(3) PL	30	3.47	0.43	3.41	0.06			(2) > (3)
Extrinsic motivation	(1) AI-VL	30	4.49	0.42	4.57	0.04	3.02	0.07	
	(2) VL	30	4.56	0.43	4.59	0.04			
	(3) PL	30	4.58	0.43	4.47	0.04			

***p < 0.001.

For intrinsic motivation, the ANCOVA results also showed a significant group difference, *F*(2, 86) = 132.59, *p* < 0.001, partial η^2^ = 0.76. The adjusted mean score was highest for the AI-VL group (adjusted M = 4.77, SE = 0.06), followed by the VL group (adjusted M = 4.02, SE = 0.06) and the PL group (adjusted M = 3.41, SE = 0.06). The partial η^2^ value indicated a large effect, showing that the learning condition had a strong effect on students’ intrinsic motivation. Bonferroni *post hoc* comparisons indicated that all three groups differed significantly from one another, with AI-VL scoring higher than VL and PL, and VL scoring higher than PL.

For extrinsic motivation, the ANCOVA revealed no statistically significant group difference after controlling for pretest scores, *F*(2, 86) = 3.02, *p* = 0.054, partial η^2^ = 0.07. The adjusted mean scores were 4.57 (SE = 0.04) for the AI-VL group, 4.59 (SE = 0.04) for the VL group, and 4.47 (SE = 0.04) for the PL group. Although the partial η^2^ value suggested a moderate effect, the group difference did not reach statistical significance. Therefore, the three learning conditions did not produce a statistically significant difference in students’ extrinsic motivation.

### 5.4 Results of cognitive load

For intrinsic cognitive load, Levene’s test was nonsignificant, *F* = 0.55, *p* = 0.58, indicating that the assumption of homogeneity of variance was met. As shown in Table 5, the one-way ANOVA showed a significant difference among the three groups, *F*(2, 87) = 72.94, *p* < 0.001, partial η^2^ = 0.63. The partial η^2^ value indicated a large effect, suggesting that learning condition explained a substantial proportion of the variance in perceived intrinsic cognitive load. The PL group reported the highest intrinsic cognitive load (M = 3.41, SD = 0.57), followed by the VL group (M = 2.59, SD = 0.52) and the AI-VL group (M = 1.79, SD = 0.48). Bonferroni *post hoc* comparisons showed that all three groups differed significantly from one another. The AI-VL group reported significantly lower perceived intrinsic cognitive load than both the VL and PL groups, and the VL group reported significantly lower perceived intrinsic cognitive load than the PL group.

**Table 5:** ANOVA results for cognitive load.

Variable	Group	*N*	Mean	*SD*	*F*	partial η^2^	*Post hoc*
Intrinsic cognitive load	(1) AI-VL	30	1.79	0.48	72.94***	0.63	(3) > (1)
	(2) VL	30	2.59	0.52			(3) > (2)
	(3) PL	30	3.41	0.57			(2) > (1)
Extraneous cognitive load	(1) AI-VL	30	2.68	0.44	9.07***	0.17	(1) > (3)
	(2) VL	30	2.72	0.58			(2) > (3)
	(3) PL	30	3.17	0.48			
Germane cognitive load	(1) AI-VL	30	3.77	0.48	42.43***	0.49	(1) > (2)
	(2) VL	30	3.31	0.44			(1) > (3)
	(3) PL	30	2.67	0.47			(2) > (3)

***p < 0.001.

For extraneous cognitive load, the homogeneity of variance assumption was also satisfied, *F* = 1.36, *p* = 0.26. The ANOVA results indicated a significant group difference, *F*(2, 87) = 9.07, *p* < 0.001, partial η^2^ = 0.17. The partial η^2^ value indicated a moderate effect, showing that learning condition had a meaningful effect on students’ extraneous cognitive load. The PL group reported higher extraneous cognitive load (M = 3.17, SD = 0.48) than the AI-VL group (M = 2.68, SD = 0.44) and the VL group (M = 2.72, SD = 0.58). Bonferroni *post hoc* comparisons showed that both the AI-VL and VL groups had significantly lower extraneous cognitive load than the PL group. However, there was no significant difference between the AI-VL and VL groups.

For germane cognitive load, Levene’s test again showed no violation of homogeneity of variance, *F* = 0.29, *p* = 0.75. The one-way ANOVA revealed a significant difference among the three groups, *F*(2, 87) = 42.43, *p* < 0.001, partial η^2^ = 0.49. The partial η^2^ value indicated a large effect, suggesting that learning condition explained a substantial proportion of the variance in germane cognitive load. The AI-VL group obtained the highest germane cognitive load score (M = 3.77, SD = 0.48), followed by the VL group (M = 3.31, SD = 0.44) and the PL group (M = 2.67, SD = 0.47). Bonferroni *post hoc* comparisons indicated that all three groups differed significantly from one another. Specifically, the AI-VL group reported significantly higher germane cognitive load than the VL and PL groups, and the VL group reported significantly higher germane cognitive load than the PL group.

## 6 Discussion

### 6.1 Discussion of physics learning achievement

Regarding Research Question 1, the results showed that the AI-VL group achieved significantly higher physics learning scores than both the VL and PL groups. The VL group also significantly outperformed the PL group.

The higher achievement of the VL group than the PL group suggests that the virtual laboratory provided effective support for learning the relationship between current and resistance. The virtual environment allowed students to manipulate variables, observe changes, and repeat experimental procedures when needed. These features may have helped students focus on the relationship between physics concepts rather than on handling physical equipment. This interpretation is consistent with Al-Duhani et al. (2023), who found that virtual laboratories improved Grade 8 students’ achievement in electricity by making abstract processes more visible and supporting repeated practice. Similarly, Finkelstein et al. (2005) showed that well-designed computer simulations could support students’ understanding of real-world physics more effectively than physical equipment in some learning contexts.

The higher achievement of the AI-VL group than the VL group further suggests that AI support provided additional benefits beyond the virtual laboratory itself. The AI agent offered timely explanations, procedural guidance, feedback, and question-answering support. These functions may have helped students correct misunderstandings, clarify experimental procedures, and connect their observations with physics concepts. This finding is consistent with Lademann et al. (2025), who reported benefits of AI chatbot support for students’ learning performance and cognitive processing. Chen and Chang (2024) also found that an AI-assisted learning environment improved students’ science learning outcomes. More importantly, the three-group comparison in the present study shows the different contributions of the two components: the virtual laboratory improved learning achievement compared with the physical laboratory, while the embedded AI agent provided further improvement beyond the virtual laboratory alone.

### 6.2 Discussion of physics learning motivation

For Research Question 2, the results showed significant differences among the three groups in overall learning motivation and intrinsic motivation after controlling for pretest scores. The AI-VL group reported the highest motivation, followed by the VL group and the PL group. However, no significant group difference was found in extrinsic motivation.

For overall learning motivation, the differences among the three groups suggest that both the virtual laboratory and the embedded AI support contributed to students’ motivation. The virtual laboratory allowed students to manipulate variables, observe experimental changes, and repeat procedures, which may have made physics learning more interactive. The AI agent provided additional explanations, procedural guidance, feedback, and question-answering support during independent learning. This timely support may have reduced students’ frustration when they encountered difficulties and helped them continue the learning task. This finding is consistent with Ait Baha et al. (2024), who found that educational chatbot support improved students’ learning experiences by allowing them to learn at their own pace, reducing stress, and maintaining motivation.

For intrinsic motivation, the higher score of the AI-VL group suggests that combining experimental exploration with immediate AI support increased students’ interest and enjoyment in physics learning. The virtual laboratory provided students with control over the learning process, while the AI agent helped them understand difficult concepts and experimental procedures. According to self-determination theory, learning environments that support autonomy and competence can promote intrinsic motivation (Ryan and Deci, 2020). In the present study, students could explore the experiment at their own pace and obtain timely explanations and feedback, which may have strengthened both autonomy and competence. This interpretation is also consistent with Becker et al. (2025), who found that AI-based support in physics learning promoted positive outcomes such as enjoyment, self-efficacy, and situational interest.

For extrinsic motivation, the nonsignificant result indicates that the three learning conditions did not produce different effects on students’ externally driven reasons for learning physics. This result differs from Chen et al. (2025), who found that a structured inquiry-based virtual laboratory improved both intrinsic and extrinsic motivation in a flipped physics classroom. This difference may be related to the design of the learning activities. The structured inquiry process used by Chen et al. (2025) included clear learning stages and task goals, which may have strengthened students’ motivation to complete the assigned activities. In the present study, the main role of the AI agent was to provide explanations, guidance, and feedback during learning, which may have had a stronger influence on students’ interest and perceived competence than on their external learning goals. Moreover, all participants were Grade 9 students from the same school, studied the same topic, and followed the same course requirements. Their extrinsic motivation scores were also relatively high across the three groups, leaving limited room for further improvement. Overall, these findings suggest that the main motivational value of AI-VL lies in strengthening students’ overall and intrinsic motivation rather than changing their external reasons for learning.

### 6.3 Discussion of cognitive load

Regarding Regarding Research Question 3, the results showed significant differences among the AI-VL, VL, and PL groups in intrinsic, extraneous, and germane cognitive load. The AI-VL group reported the lowest intrinsic cognitive load and the highest germane cognitive load. Both the AI-VL and VL groups reported lower extraneous cognitive load than the PL group.

For intrinsic cognitive load, the AI-VL group reported the lowest score, followed by the VL group and the PL group. This result should be interpreted as a difference in students’ perceived intrinsic cognitive load because the scale measured their perceptions of content complexity. According to cognitive load theory, intrinsic cognitive load is mainly influenced by task complexity, element interactivity, and learners’ prior knowledge (Sweller et al., 2019). The AI agent did not change the objective complexity of the physics content. Instead, its explanations, hints, and feedback may have helped students organize the task elements and understand the relationship between current and resistance more effectively. This interpretation is consistent with Chen and Chang (2024), who found that AI-assisted science learning reduced students’ perceived mental burden. Thus, the lower intrinsic cognitive load in the AI-VL group may reflect students’ improved ability to manage the complexity of the learning task.

For extraneous cognitive load, both the AI-VL and VL groups reported significantly lower scores than the PL group, while no significant difference was found between AI-VL and VL. This result suggests that the virtual laboratory itself reduced unnecessary cognitive demands. In the PL condition, students needed to connect wires, adjust equipment, read instruments, avoid operational errors, and manage physical laboratory constraints. These procedures may have required cognitive resources that were not directly related to understanding the relationship between current and resistance. In contrast, the virtual laboratory provided a structured and visually guided environment that reduced unnecessary operational demands. This result is consistent with Thees et al. (2020), who found that students in a technology-supported physics laboratory reported lower extraneous cognitive load than those in a traditional laboratory. They explained that presenting relevant information close to the experimental equipment reduced unnecessary visual search and information processing.

The non-significant difference between AI-VL and VL also shows that adding the AI agent did not create additional extraneous cognitive load. This result differs partly from Lademann et al. (2025), who found that AI-generated explanations produced lower extraneous cognitive load than traditional textbook materials. The difference may be related to the comparison conditions. Lademann et al. compared AI-generated explanations with textbook materials, whereas both the AI-VL and VL groups in the present study used the same structured virtual laboratory. The VL may already have reduced unnecessary cognitive demands, leaving limited room for the AI agent to produce a further reduction. Nevertheless, the additional dialogue, feedback, and question-answering functions did not increase students’ extraneous cognitive load.

For germane cognitive load, the AI-VL group reported the highest score, followed by the VL group and the PL group. This result suggests that AI-VL encouraged students to invest more cognitive effort in understanding and organizing the learning content. The AI agent may have supported this effort by guiding students to interpret experimental results, clarify conceptual relationships, and connect their observations with Ohm’s law. The VL environment also produced higher germane cognitive load than the PL condition because students could repeat operations and focus on the relationships among variables. This interpretation is supported by Alam et al. (2026), who suggested that AI-supported learning can improve learning outcomes by helping students manage cognitive demands and direct their effort toward meaningful learning. Overall, the findings suggest that the virtual laboratory mainly reduced unnecessary operational demands, while the AI agent helped students direct more cognitive effort toward understanding the physics concepts without adding extraneous load.

## 7 Implications

This study has several theoretical implications. First, it extends research on virtual laboratories by showing that VL can support physics learning more effectively than PL for a topic involving abstract and invisible processes, such as the relationship between current and resistance. Second, it adds evidence to AI-supported learning by showing that an AI agent can be embedded within a virtual laboratory to support flipped physics learning, rather than being used as a separate tutoring tool. The results suggest that AI-based explanations, experiment guideline, feedback, and question-answering support can help students address difficulties during independent learning. Third, the findings contribute to cognitive load theory by showing that AI-VL reduced intrinsic cognitive load and increased germane cognitive load without increasing extraneous cognitive load compared with VL. This indicates that well-designed AI support may help students manage task difficulty and invest more cognitive effort in meaningful learning.

This study also provides practical implications for physics teaching. Teachers can use AI-supported virtual laboratories to strengthen flipped learning, especially when students need to conduct experimental exploration before class. Compared with videos and textbooks alone, AI-VL allows students to manipulate variables, observe experimental changes, repeat procedures, and receive timely learning support. The findings also suggest that AI agents should be designed as learning guides rather than answer providers. In this study, the AI agent was trained on Grade 9 physics content, restricted to physics-related responses, and designed to guide students’ thinking instead of giving final answers. Schools may therefore consider AI-VL as a complementary approach to physical laboratories, particularly for pre-class preparation, review, and physics topics in which key processes are difficult to observe directly.

## 8 Limitations and future directions

This study has several limitations that should be addressed in future research. First, the sample included 90 Grade 9 students from one middle school in central China, so future studies could include larger samples from different schools and regions to examine the generalizability of the findings. Second, this study used different teachers across the three experimental conditions, which may have introduced potential teacher-related effects. Future studies could use the same teacher across conditions, when feasible, to improve experimental rigor. Third, the intervention lasted for 3 weeks, which was sufficient for examining short-term effects but not for determining whether students’ achievement, motivation, and cognitive load would remain stable over time. Future research could conduct longer interventions and include delayed posttests to examine whether the effects of AI-VL can be maintained. Fourth, this study focused on one physics topic, namely the relationship between current and resistance under constant voltage in Ohm’s law, and the features of this topic may be especially suitable for virtual experimentation and AI-supported guidance. Future studies could apply AI-VL to other physics topics or other STEM subjects. Lastly, this study did not examine students’ specific interaction patterns with the AI agent, such as the number of questions asked, types of prompts used, dialogue quality, or feedback frequency. Future research could collect log data, dialogue records, and learning behavior data to identify which forms of AI interaction are most closely related to achievement, motivation, and cognitive load.

## 9 Conclusion

This study examined the effects of an AI-supported virtual laboratory on middle school students’ flipped physics learning. Using a three-group quasi-experimental design, the study compared AI-VL, VL, and PL in terms of physics learning achievement, learning motivation, and cognitive load. The results showed that AI-VL produced the strongest learning achievement and motivation, especially by improving students’ overall learning motivation and intrinsic motivation. AI-VL also reduced intrinsic cognitive load and increased germane cognitive load, while the addition of the AI agent did not increase extraneous cognitive load compared with VL. These findings suggest that integrating AI into a virtual laboratory can provide timely explanations, guidance, feedback, and question-answering support during independent flipped learning. Therefore, AI-VL can be considered a useful approach for supporting middle school students’ physics learning, particularly for topics that require students to connect abstract concepts with experimental observations.

## Data Availability

The raw data supporting the conclusions of this article will be made available by the authors, without undue reservation.
